# Engineering biology applications for environmental solutions: potential and challenges

**DOI:** 10.1038/s41467-025-58492-0

**Published:** 2025-04-14

**Authors:** David J. Lea-Smith, Francis Hassard, Frederic Coulon, Natalie Partridge, Louise Horsfall, Kyle D. J. Parker, Robert D. J. Smith, Ronan R. McCarthy, Boyd McKew, Tony Gutierrez, Vinod Kumar, Gabriella Dotro, Zhugen Yang, EBIC partners, David J. Lea-Smith, Francis Hassard, Frederic Coulon, Louise Horsfall, Kyle D. J. Parker, Robert D. J. Smith, Ronan R. McCarthy, Boyd McKew, Tony Gutierrez, Vinod Kumar, Gabriella Dotro, Zhugen Yang, Thomas P. Curtis, Peter Golyshin, Sonia Heaven, Bruce Jefferson, Paul Jeffrey, Davey L. Jones, Kristell Le Corre Pidou, Yongqiang Liu, Tao Lyu, Cindy Smith, Alexander Yakunin, Yue Zhang, Natalio Krasnogor, Natalio Krasnogor

**Affiliations:** 1 University of East Anglia, Norwich, UK; 2 Cranfield University, Cranfield, UK; 3 GitLife Biotech Ltd, Newcastle Upon Tyne, UK; 4 University of Edinburgh, Edinburgh, UK; 5 Brunel University London, Uxbridge, UK; 6 University of Essex, Colchester, UK; 7 Heriot-Watt University, Edinburgh, UK; 8 Environmental Biotechnology Innovation Centre, Cranfield, UK; 9 Newcastle University, Newcastle upon Tyne, UK; 10 Bangor University, Gwynedd, UK; 11 University of Southampton, Southampton, UK; 12 Cranfield University, Bedford, UK; 13 University of Glasgow, Glasgow, UK

**Keywords:** Environmental biotechnology, Applied microbiology, Molecular engineering, Bioremediation, Governance

## Abstract

Engineering biology applies synthetic biology to address global environmental challenges like bioremediation, biosequestration, pollutant monitoring, and resource recovery. This perspective outlines innovations in engineering biology, its integration with other technologies (e.g., nanotechnology, IoT, AI), and commercial ventures leveraging these advancements. We also discuss commercialisation and scaling challenges, biosafety and biosecurity considerations including biocontainment strategies, social and political dimensions, and governance issues that must be addressed for successful real-world implementation. Finally, we highlight future perspectives and propose strategies to overcome existing hurdles, aiming to accelerate the adoption of engineering biology for environmental solutions.

## Abstract (web summary)

The scale of global environmental challenges requires a multi-pronged approach, which utilises all the technologies at our disposal. Here, authors provide their perspective on the potential of engineering biology for environmental biotechnology, summarizing their thoughts on the key challenges and future possibilities for the field.

## Introduction

Addressing environmental challenges arising from growing, increasingly industrialised global populations and urbanisation will rely on various technologies, including engineering biology. Engineering biology is applicable to the detection and degradation of pollutants, greenhouse gas sequestration, and conversion of waste streams, especially recalcitrant and non-biodegradable ones, to value-added product generation and replacement of fossil fuel-derived production with biological alternatives^1^. The UK government defines engineering biology as ‘the design, scaling, and commercialisation of biology-derived products and services that can transform sectors or produce existing products more sustainably’ and has designated the field as one of five critical technologies^2^. Engineering biology is sometimes synonymous with synthetic biology or biological engineering, although in this case, the goal is to take synthetic biology concepts and translate them into practical solutions to address real-world issues and markets and, at the same time, create value chains out of them. Combined private and public investment in the synthetic biology sector totalled US$16.35 billion in 2023, with the market size expected to reach approximately US$148 billion by 2033^3^.

This article focuses on the potential applications, considerations and challenges of utilising and engineering microbes for environmental applications, which we term environmental biotechnology. We do not focus on the synthetic biology tools, except where they impact environmental implementation, biocontainment, biosafety, or regulatory requirements. There are significant obstacles that need to be overcome to fully realise the potential of environmental biotechnology^4^. Scaling-up these engineered systems and their digital twins to real-world applications needs better understanding to bridge the gap between lab conditions and complex environments^5,6^. Developing suitable standards^7^ and addressing ethical, regulatory, and societal dimensions, play significant roles in deploying synthetic biology applications^8^. These considerations will be discussed in greater detail in the following sections with relevant examples provided.

## Application-specific innovations and challenges

**Fig. 1:**
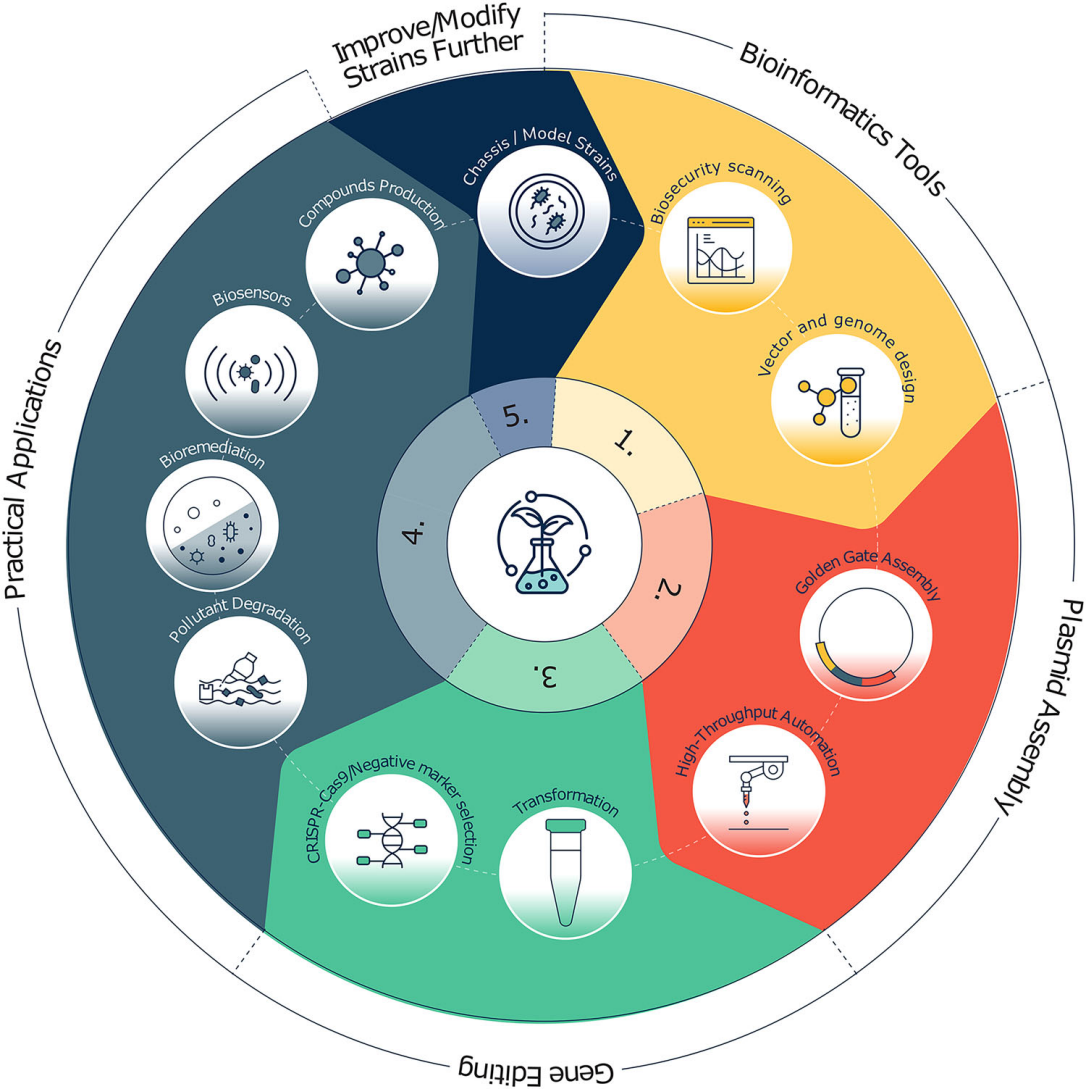
Overview of synthetic biology tools and techniques for environmental applications.

Engineering biology relies on a range of technologies, including synthetic biology tools for rapid plasmid assembly and precise chromosomal modification, which can be upscaled via automation^8^ (Fig. 1). A recent report from Barclays listed 379 active engineering biology companies in the UK, but few focus on environmental solutions. Supplementary Data 1 lists some companies working in engineering biology and/or bioremediation^9,10^. Companies can be grouped by environmental application and the shared challenges and opportunities this presents.

Companies convert greenhouse gases into valuable compounds using phototrophic organisms such as cyanobacteria, algae, or plants (e.g., CyanoCapture, Biorizon Biotech, Algenol, Cemvita Factory, Checkerspot, Aequor) or metabolically engineered heterotrophic bacteria (Lanzatech, Mango Materials, Newlight Technologies). Income is derived from carbon permits and the final product. Carbon permit and/or Emission Trading Schemes (ETS) prices vary between countries and over time (i.e., since 2022, from 50 to 100 euros per tonne in the EU), with global revenues reaching US$104 billion in 2023. However, at these prices, most income will have to be derived from the final product. Over 50 products, mainly low-value ones like bioplastics and biofuels, have been generated from gaseous feedstocks. This greenhouse gas-based production can partially replace fossil-based manufacturing, mitigate climate change, and contribute to delivering net-zero emissions^11,12^. However, commercial success is challenging unless petroleum or carbon permit prices increase or higher-value products are generated. Political challenges, like higher industry costs and potential job losses, make increasing carbon permit prices difficult. Greater parity for carbon permits and ETS between countries may aid political implementation.

Companies using plant-derived sugars for the production of industrial compounds (Genomatica), textiles (Modern Synthesis, Solena Materials), pigments (Colorifix, Pili), or bioplastics (Danimer Scientific, Genecis, Pond, Intropic Materials, EVA Biosystems, Biome Bioplastics) compete with petroleum-derived products and would also benefit from these policies. One issue these companies face is competition with food production and limited agricultural land, which restricts the scaling up of commercially viable processes. For example, Brazilian ethanol production from sugar cane has remained stable, with minor increases primarily due to greater land usage linked to deforestation^13^, which is spatially and environmentally unsustainable. Lignocellulosic-based bioethanol production has been minimal but could increase yields, with commercially viable production demonstrated by Razien SA. Utilising lignocellulose biomass to produce higher-value compounds, like food substitutes (e.g., Supplant, Novonutrients), may be more economically viable and limit concerns about food competition. Companies using engineered microbes to improve soil quality, crop yields, and reduce fertiliser use (Pivot Bio, Pantego) could help alleviate this issue.

The environmental remediation market is valued at approximately US$115 billion (Grand View Research), with many companies offering a wide range of in-situ or ex-situ remediation strategies, including physical, chemical, and biological remediation of soils, water (e.g., Microvi), brownfield sites or industrial wastes. Growth is driven by increasing regulatory frameworks and policies to reduce environmental pollutants such as PAHs (polycyclic aromatic hydrocarbons), PCBs (polychlorinated biphenyls), PFAS (Per- and polyfluoroalkyl substances), plastics, pharmaceuticals, pesticides, and heavy metals/metalloids. Many remediation companies (e.g., AST Environmental, Probiosphere, Drylet, In-Situ Remediation Services, VHE, UK Remediation, Veolia Remediation Services, Soilfix Ground Risk Solutions, Sumas Remediation Service) offer bioremediation among their services, and the market is forecast to be $17.8 billion by 2025 and growing at a predicted rate of over 10% per year. Bioremediation strategies include biostimulation of native microorganisms or plants with the addition of nutrients, oxidants, electron donors/acceptors, and biosurfactants, and controversially, the addition of microbes with the genetic capacity to biodegrade target pollutants, which may have limited effectivity compared to native communities^14^. There are few commercial bioaugmentation products, which typically contain only undisclosed and unmodified Class I organisms. Despite much research interest since the 1980s^15^, and some successes in modifying bacteria with enhanced biodegradation capacities^16,17^, there remain no commercial applications of engineered microbes for bioremediation. This can be attributed to difficulties in engineering microbes that can outcompete native organisms while only targeting recalcitrant pollutants representing a tiny fraction of the available organic carbon pool, as well as a lack of field trials, regulatory hurdles, and safety and containment concerns about releasing GMOs.

Another avenue is to engineer microbes to produce products, such as biosurfactants, that can be used in remediation. The global biosurfactants market exceeded USD $1.5 billion in 2019 and is projected to grow at over 5.5% from 2020 to 2026^18^ with multiple manufacturers, including Ecover, Jeneil Biotech, Evonik, and Biotensidon^19^. The increasing global interest in biosurfactants is due to their low toxicity, biodegradability, low environmental footprint, and impact^20^, though they are typically more expensive than synthetic chemical surfactants^21^. Household detergents are the largest application market, followed by cosmetics and personal care, and the food industry^19^. Whilst there are some remediation products that contain biosurfactants for small-scale pollution bioremediation (e.g., Motivate Biostimulant, Bio8 Industrial), the major challenge is upscaling production to replace for example, the chemical surfactants used within oil spill dispersants, while keeping costs low. Production of biosurfactants at scale remains challenging even with improved production strategies^19^ and engineering biology approaches^22–24^.

## Integration with other technologies

**Fig. 2:**
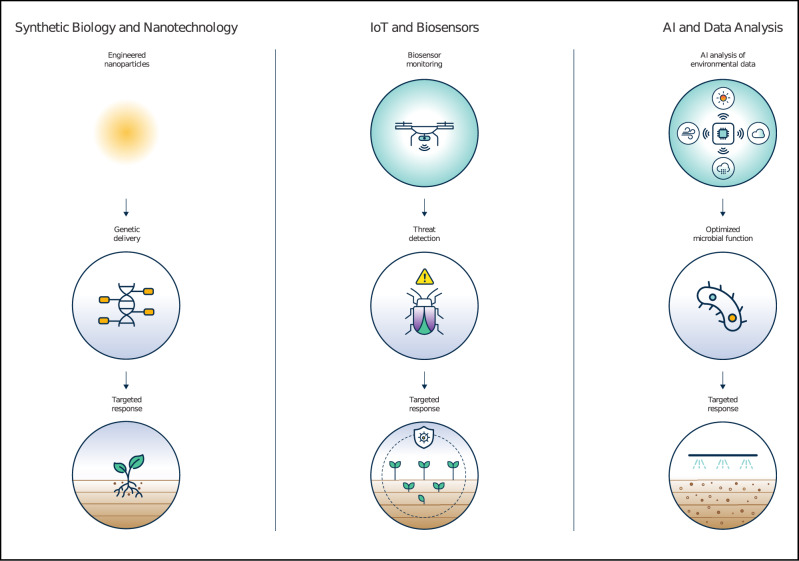
Integration of synthetic biology with emerging technologies for enhanced environmental monitoring and remediation.

Integrating synthetic biology with nanotechnology, the Internet of Things (IoT), and artificial intelligence (AI) enhances deployment of engineering biology for environmental applications^25^ (Fig. 2). Synthetic biosensors, including cell-based and cell-free devices^26^, can detect a wide range of target molecules such as pollutants, heavy metals and biomarkers with high precision and reliability. These biosensors are usually low cost, easy to use and can operate in remote or resource-limited settings, making them ideal for integration into environmental monitoring systems via IoT and AI. The synergy between synthetic biology and the IoT is transforming environmental monitoring. IoT devices with sensors and sensor network can track environmental conditions in real-time, triggering genetically engineered microbes to respond to detected pollutants by activating specific metabolic pathways. This adaptive response is useful in dynamic environments where rapid changes in conditions, such as fluctuating pollutant levels, could require a directed response or action. For instance, microbes could increase the production of enzymes to degrade toxins, signal for remediation measures, or adjust their activity to optimise pollutant removal^27^. AI could complement this by analysing vast amounts of environmental data to predict the behaviour of bioengineered organisms under a variety of conditions, enabling the optimisation of their functions in complex ecosystems. This application is particularly relevant in tasks such as biodegradation and carbon capture, where tailored organism functions could significantly increase efficiency^28,29^.

Furthermore, the integration of synthetic biology with robotics has led to automated bioreactors that maintain optimal conditions for engineered organisms. These robotic systems regulate factors such as temperature, pH and nutrient flows, ensuring consistent growth and activity levels. Automation also streamlines sampling and testing processes, enabling continuous monitoring crucial for large-scale applications^30^. The convergence of synthetic biology with cyber-physical systems, including digital twins, opens new frontiers in environmental management^31^. Interconnecting these systems via the IoT enables the collection of real-time, geographically spread data on environmental parameters, which AI can utilise to predict environmental trends and adjust the behaviour of bioengineered organisms accordingly. Additionally, geospatial technologies combined with synthetic biology enhance multi-scale spatial management of biotechnological interventions. Unmanned aerial vehicles, equipped with enhanced biosensors could be applied to identify areas heavily impacted by pollutants, guiding targeted deployment of genetically engineered plants or microbes where they are most effective^32^. Cloud computing and cloud laboratories manage the vast data generated from synthetic biology applications, allowing refinement of design and deployment strategies to ensure effectiveness and safety at large scales^33^. Bio-cyber factories are integrated systems combining biological production with cyber-physical components, allowing for decentralised and automated bio-manufacturing processes. They utilise real-time data and computational models to optimise biological production, potentially contributing to decentralised bio-production and addressing rural-urban migration and gender inequality^24^.

Finally, hybrid engineered living materials (HELM) is an emerging field that blends living organisms, typically microorganisms, with inert substrates to forge materials with enhanced and novel functionalities. This innovative approach leverages the principles of synthetic biology to imbue these composites with capabilities such as in-situ sensing and the bioremediation of pollutants, including heavy metal ions^34^. Additionally, HELM has facilitated the development of biocomposite thermoplastic polyurethanes. These materials were embedded with *Bacillus subtilis* spores genetically tailored through both adaptive laboratory evolution and synthetic biology techniques. Such engineering allows these spores to withstand the high temperatures required for polyurethane production and includes a fluorescent reporter to monitor spore germination^35^. This integration of living materials into synthetic matrices represents a significant advancement in materials science, combining biological functionality with traditional engineering materials to address environmental challenges^36^.

## Commercialisation and scale-up of engineering biology

Commercialisation of engineering biology has proven challenging, partly because it is a relatively new suite of technologies. A relevant comparison is with solar, wind, and nuclear energy, which had decades of public and private investment in fundamental sciences and translation to practical solutions before full deployment. That sustained investment moved upwards the technological readiness level of the science & technology behind those disciplines. Careful resource investment works and delivers results to society. Therefore, engineering biology would benefit from sustained investment to demonstrate application at scale. Another factor that could improve commercialisation is standardising legislation between major markets on what types of engineered organisms can be deployed. This is especially critical if organisms are to be released in open environments^37^. Achieving commercial success is harder for technologies limited to certain countries. The regulatory distinction is an arbitrary one and should not be focused on how the organism was made but rather on what it does. For example, there is no fundamental distinction between a markerless engineered organism if it is modified via CRISPR or using selectable markers like *sacB*. Given the scale of our environmental challenges, this regulatory distinction could hamper environmental protection by fostering inactivity.

Downstream processing, encompassing the separation and purification of the desired product from the culture medium, poses significant challenges at scale^38^. Steps such as centrifugation, filtration, and chromatography must be optimised to maintain high yield and purity while managing increased material volumes. How well genetic constructs persist during scale-up is another vital consideration. Engineered strains should display genetic stability and perform under industrial conditions, ensuring desired traits are maintained over extended production cycles and larger volumes^39^. Contamination control becomes more challenging as the scale increases. Implementing effective sterilisation procedures and designing bioreactors to minimise contamination risks are essential for maintaining axenic cultures, thereby ensuring the competitiveness and productivity of engineered strains^40^. Scaling-up and integrating Engineering Biology technologies into real-world settings also requires practitioner knowledge, which should be accessed at the conceptualisation and design phases so that it can shape decision-making from the outset. Progressing the field is therefore reliant on learning from past pitfalls, assessing why specific processes were unsuccessful or not commercially viable, which are often unreported by companies. Disclosure of data, including scaled-up outcomes post-IP protection, is crucial and arguably should be mandatory for entities receiving government funding. While privately funded companies may hesitate to publish, sharing such information could be beneficial, particularly for scaling processes optimised in the laboratory. Despite potential risks of unauthorised technology use, this transparency allows scrutiny by the scientific community, leveraging peer review as expert consultation. Suggestions from such scrutiny could enhance processes, potentially minimising investor losses on non-viable technologies or enhancing commercial prospects for viable ones. Moreover, this approach can aid in retaining top research talent motivated by scientific publication, akin to the successful model pioneered by Genentech^41^.

Economic viability is crucial for scale-up, requiring process optimisation to reduce costs associated with energy, raw materials, and labour while maximising yield and productivity. Economic modelling can improve cost-efficiency predictions. Regulatory compliance is essential, ensuring processes meet standards for environmental safety, product quality, and operational practices. Achieving compliance is critical for market approval and the commercial success of the biotechnology solution^42^. Beyond the bioreactor, deploying synthetic biology in environments where engineered organisms face competition requires additional consideration. In such systems, engineered microbes must maintain their functional roles amidst native microbial communities. Strategies include developing synthetic microbial consortia to outcompete natural populations, designing genetic constructs that enhance resilience and adaptability, and incorporating mechanisms for dynamic interaction with the environment^43^. For instance, engineered microbes might be programmed to switch metabolic pathways in response to environmental signals, maintaining activity under fluctuating conditions. Genetic stability of strains is less important for biosensors and bioremediation utilised once or over short periods of time and could even be advantageous. For example, multiple studies have successfully used microbial consortia specialised for hydrocarbon degradation to bioremediate contaminated soil^44^, and it is unlikely that such organisms persist in a hydrocarbon-deficient environment. Bioremediation of sites polluted with heavy metals or PFAS can restore habitats, facilitate the return of native species, and improve ecosystem health^45^. Other challenges and opportunities in applied synthetic biology are discussed in Hanson and de Lorenzo, 2023^46^.

## Biosafety and biosecurity

Engineering processes for genetically modified microorganisms and their use in environmental biotechnology must be safe and trustworthy. Deploying engineered microbes in the environment needs thorough ecological risk assessments to prevent adverse effects on biodiversity. Strategies that enhance microbial efficiency must be balanced with safeguards to avoid unintended ecological consequences, such as disrupting native microbial communities or affecting higher trophic levels. The UK has strict legislation controlling the deliberate release of genetically modified organisms (GMOs). Since 2011, only three consents for GMO release have been granted, with two pending. In the US, three agencies are responsible for regulating GMOs, the Food and Drug Administration, the Department of Agriculture, and the Environmental Protection Agency. It is likely that any microbes utilised for environmental solutions would be regulated by the latter two. In the European Union, the regulatory framework for GMOs is undergoing revisions to account for new genomic techniques (NGTs). The European Commission is considering updates that may differentiate between organisms produced by conventional genetic modification and those developed through precise gene-editing methods, potentially easing restrictions for certain environmental biotechnology applications.

The development of technologies for precise genetic modification of plants allowed for the passing of the Genetic Technology (Precision Breeding) Act in the UK in 2023, similar to legislation already in place in Argentina, the US, Australia and Japan. The act distinguishes GM from precision breeding in that the former may include genetic changes that could not have occurred naturally through traditional breeding. As such, the act covers precision-bred plants and may extend to animals engineered with gene-editing techniques. The availability of similar technology for microbes means such acts should be extended to cover the use in environmental biotechnology. Controlled environmental release of microbes engineered with antibiotic resistance genes is highly unlikely, as these may transfer to pathogenic species. However, technologies to precisely gene edit microbes mean it is possible to produce strains containing no additional foreign DNA. Selectable markers, including *sacB*, allow removal of antibiotic resistance genes. Increasing application of CRISPR-Cas to a broader range of microbes, coupled with Directed Accelerated Revision Technology, opens new opportunities and challenges for environmental synthetic biology biosafety and biosecurity^47–49^.

The current state of biosafety and biosecurity standards related to environmental biotechnology both overlaps with and leaves gaps in relation to other regulations^50^. Well-established guidelines exist for traditional biotechnological applications (contained use). For most application types, the risk assessment for genetically modified or edited organisms will account for the nature of the intended modification and will assess the relative risk to the environment, and animal or human health (e.g., hazards, severity, likelihoods, etc.). Containment level (BSL1 to BSL4), safeguards, control measures, and assignment of GM Activity Class require consideration. However, as we venture into more advanced areas like synthetic biology and gene editing, existing frameworks often fall short, especially when considering the potential of these technologies to bring solutions that may require deliberate release.

**Fig. 3:**
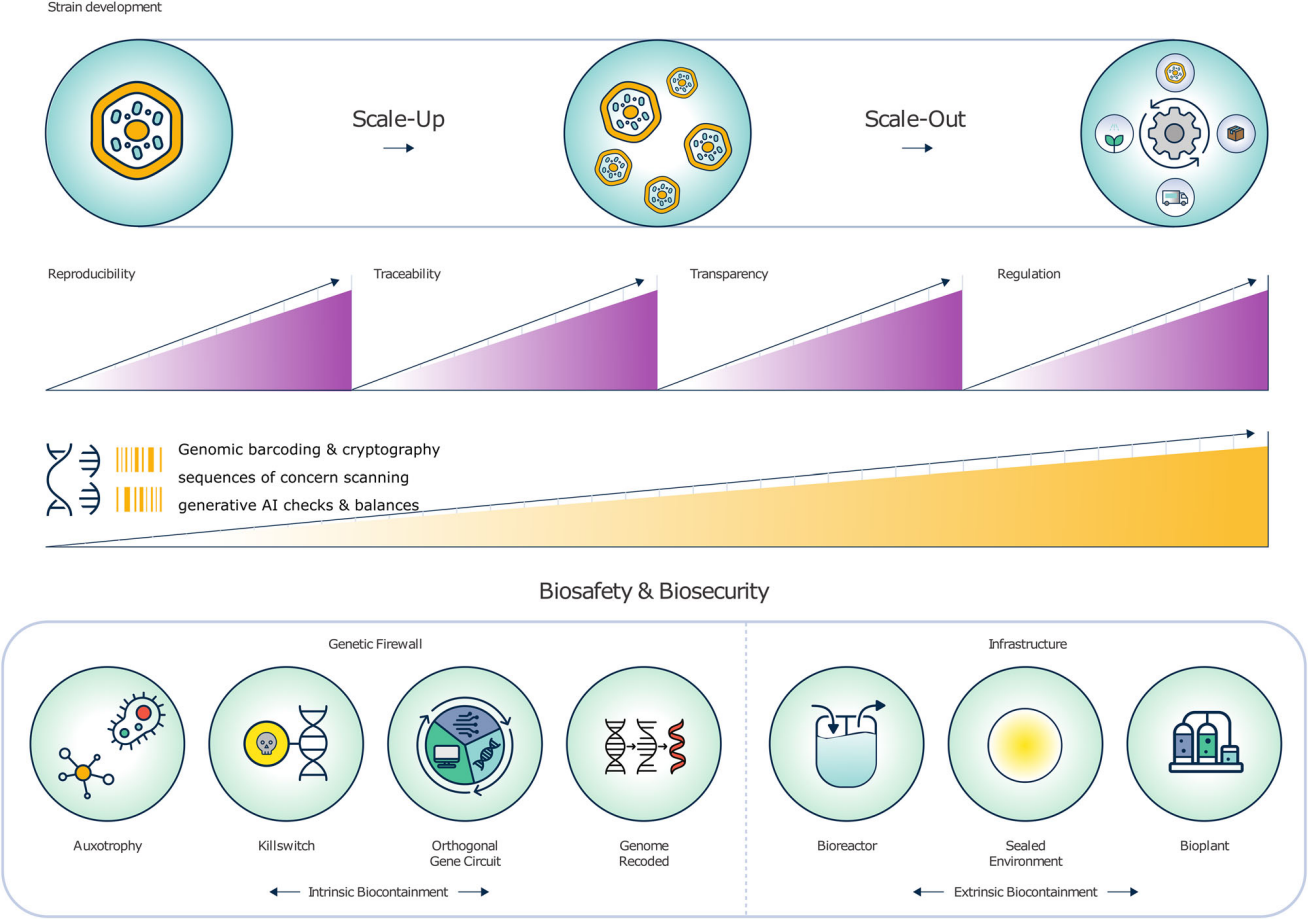
Strategies for biosafety and biocontainment in environmental biotechnology.

Important technical challenges remain in terms of containment strategies and procedures and control measures (Fig. 3). An engineered organism that might be released into the environment accidentally, maliciously or with positive intent may bring risks that need to be managed. These include (a) the potential of the engineered organisms to “out-fitness” the native microbiome, (b) that the engineered organisms might not perform as intended with a phenotype that, while optimal to the target environment, might be detrimental in a different one, (c) that a trait of the engineered organism might unintentionally spread through the native microbiome by, e.g., horizontal gene transfer. To address these risks, biocontainment strategies have been proposed to improve biosafety of microorganisms^51^. There are three main routes to genetically encoded biocontainment, meaning modified organisms are intrinsically bio-contained rather than physically contained within flasks, reactors, or other facilities. These strategies are sometimes referred to as genetic firewalls. One could bio-contain at the DNA replication level by modifying the DNA replication machinery of the organism to include some level of auxotrophy for some essential compound(s), which could be natural or new-to-nature nucleotides, or via the development and dependence on entirely orthogonal DNA replication enzymes or synthetic amino acids. At the transcriptional level, biocontainment strategies could involve synthetic gene circuits, orthogonal RNA polymerase-promoter pairs, and other regulatory interventions. At the translation level, strategies can focus on auxotrophy for natural amino acids, non-canonical amino acids and modified ribosomes^52^. Although research on the use of non-canonical nucleotides and non-canonical amino acids is growing, there are many open questions and unexplored areas of potential synergy with biosecurity and biosafety. At the time of writing, there are many non-canonical nucleotides known, but only a few have been explicitly used as biosafety or biosecurity tools to enhance biocontainment and genetic firewalls^53^. An overview of different potential biocontainment approaches is available through resources like the Biocontainment Finder (https://standardsinsynbio.eu/biocontainment-finder/) which catalogues various strategies and their effectiveness.

It is now commonly accepted that genetically encoded biocontainment escape rates must remain below 10^−^^8^ and ideally much lower to be of practical relevance^54^. Escape rates refer to the probability or frequency at which a GMO might escape its intended containment measures, more precisely, the likelihood of a single organism escaping containment per generation (or per replication event). Reported escape rates are based on laboratory-scale experiments with limited cell numbers. Scaling up to industrial volumes introduces additional variables and uncertainties. Therefore, while current data suggest low escape rates, these figures may not directly extrapolate to large-scale applications, highlighting the need for further research. Several studies have reported, using various approaches (e.g., orthogonal gene circuits, pH-sensitive kill switches, and engineered auxotrophy), lower escape rates, which via a combined strategy reached 10^−^^12^ for *E. coli*^55^. Escape rates of 10^−^^13^ by developing an auxotrophic system in which *E. coli* required inorganic phosphate to be exogenously supplied^56^ and 10^−^^6^ to 10^−11^ in *Saccharomyces cerevisiae*, via a layered biocontainment strategy, have been achieved^57^. Arnolds et al. argued that existing laboratory demonstrators of biocontainment might still be inadequate for realistic industrial-scale bioproduction, let alone environmental release^58^. Laboratory and pilot scales range from 0.5 to 10 and 50 to 200 L, respectively, while industrial-scale ranges from 500 to 5000 L. Therefore, a fed-batch bioreactor reaching cell densities of 10^10^ cells/L with a 1000 L capacity would have at any one time, 10^13^ cells and hence one could expect escape rates of 10^*x*^, with *x* > 1, cells per generation. Much larger facilities are being built; for example, Samsung Biologics will have a biomanufacturing capacity of 784,000 L, rendering existing proof-of-concept genetically encoded biocontainment strategies potentially insufficient. Moreover, fully recoded genomes, i.e., genomes that had certain codons eliminated from the translation machinery, reassigned to a different amino acid or to the ‘stop’ instruction, which could in principle provide lower escape rates for biocontainment, remain expensive, time-intensive, and impractical for most applications.

Notwithstanding recent progress and theoretical work^59,60^ on “ecological firewalls” in which ecological networks and species diversity act as a containment mechanism, it remains uncertain whether any individual measure or combination thereof can achieve failproof genetic firewalls ensuring zero propagation of recombinant DNA or strains. Given the inherent limitations of genetically encoded containment strategies and the arguments to deploy engineered biological systems in open environments, a complementary approach to containment that emphasises traceability is also being pursued. Traceability is facilitated by unique identifiers stably integrated into genomic regions. Wang and Zhang recommends^61^, as complementary strategies to biocontainment, the utilisation of genomic barcodes or watermarks^62^. The European Food Safety Authority (EFSA) scientific committee has recommended that engineered microbes be barcoded^63^. In the absence of genomic barcodes, tracking engineered biological assets requires full genome sequencing and analysis with sophisticated machine learning tools^64^, usually trained on proprietary data sets, to ascertain the origin of an engineered organism or of a recombinant gene sequence. This shift is facilitated through use of unique identifiers stably integrated into permissive genomic regions. Furthermore, very recent advances have enabled the traceability of horizontal gene transfer events via barcoded ribozymes that are inserted into mobile genetic elements such as plasmids or transposons^65^. The absence of a streamlined system to pre-emptively identify the provenance of bioassets or their key biosafety attributes, e.g., Generally Recognised As Safe (GRAS) and Qualified Presumption of Safety, propensity for horizontal gene transfer both as recipient or donor, etc^66^, hinders the scalability of engineering biology and increases the potential for error. This challenge may limit the growth and acceptance of bio-based products in the market or release into the environment. Therefore, we have argued^4^ that physically linking engineered strains to their digital twins via genomic barcodes would enhance transparency, collaboration, and traceability of biological assets^67^, while contributing to more clarity and friction reduction on the route to securing regulatory approvals^66^.

In any case, given the current state-of-the-art on environmental biotechnology we have identified the following governance challenges:

- Technological complexity: Biotechnology’s growing complexity makes it hard to create universal standards, specifically universal biocontainment standards. Scalable solutions across laboratory, pilot, and industrial scales would be necessary for biocontainment to become a viable strategy. To help bridge this gap, a more pragmatic approach built around traceability of genomic barcodes, DNA-based error-correcting codes^68^ emerging within the field of DNA data storage and nucleic acids-focused cryptography^69^, that are supported with existing technologies accessible to most laboratories and industries, can provide sufficient assurance to scientists, industry, regulators and the public at large.
- Global Consistency: Different nations maintain distinct regulations and standards, complicating the regulatory landscape for global biotechnology firms and academics alike. Biosafety and biosecurity matters must be dealt with in a more integrated and seamless manner than currently practiced.
- Data Privacy, Security and IP rights: As biotechnology becomes more data-driven and amalgamates with automation and AI, it will further accelerate and hence, safeguarding biological data, privacy and IP are a significant concern.

Thus, a more dynamic, adaptable approach to biosafety and biosecurity for environmental biotechnology built around screening for sequences of concern (SoC) and complementary approaches of biocontainment (for prevention of harm) and traceability via, e.g., genomic barcoding and cryptographic schemes (for identification, liability and IP protection) is needed, ideally, with the development of suitable supporting standards, metrics, and informatics infrastructure. Moreover, the potential use of adversarial generative AI in, e.g., the retrobiosynthesis of enzymatic pathways for toxins^70^, the design of non-homology detectable AMR and, more generally, the detection of engineered modifications whether malicious or not, necessitates urgent and vigorous research as well as international coordination. A recent example of effective international cooperation and rapid coordination in this area is the establishment of the USA-UK biosecurity dialogue^71^ and their alignment of UK^72^ and USA^73^ DNA synthesis biosecurity screening guidelines for SoCs, which will apply to all providers, intermediaries, and end users of synthetic nucleotide sequences. There are auspicious first steps in this direction, e.g., the International Biosecurity and Biosafety Initiative for Science (IBBIS) provides the open-source common mechanism^74^, while SecureDNA^75^ and open and verifiable DNA scanning software stack and services.

## Social, political and value dimensions

**Fig. 4:**
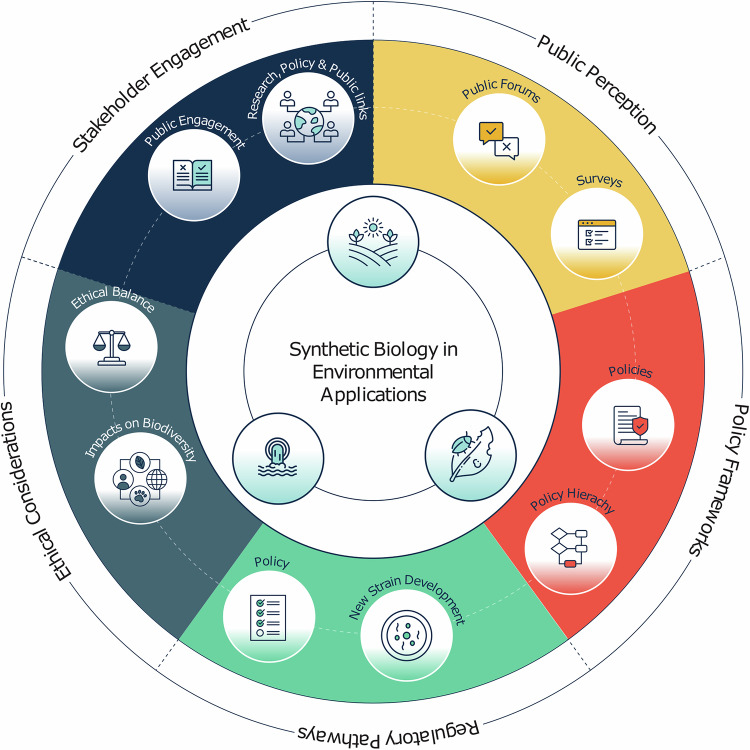
Social and policy dimensions for synthetic biology for environmental applications.

Addressing environmental goals through engineering biology is as much a social, political and economic challenge as a technical one. It is therefore necessary to incorporate socio-technical knowledge into the design of any environmental biotechnology solution (Fig. 4)^76–78^.

The nature of environmental problems poses two challenges for environmental biology solutions, where socio-technical knowledge can help. First, their complexity means experts may disagree about the causes of a problem and whether, and how, it can be addressed with a technological solution. For instance, the problem of municipal water contamination and pollution is clearly due to the technological system of water treatment, but the functioning of that system also significantly depends on the regulatory, political, and economic decisions of people in and around it^79^. Second, because these problems are multifaceted, addressing one aspect can cause knock-on effects elsewhere. For example, EU policies to increase biofuel production led to habitat and biodiversity losses due to land use changes, particularly loss of rainforests for palm oil plantations, and increased prices for staple food crops^80,81^. Therefore, it is imperative to question how engineering biology can effectively address environmental challenges without inadvertently creating new challenges or amplifying existing social and political disparities due to the introduction of novel technologies^82,83^. Moreover, as the technology evolves, it is crucial that synthetic biology developments are managed responsibly throughout their lifecycle^8^. New approaches to Life Cycle Analysis can aid decision-making, particularly if assumptions, such as defining system boundaries (what is included or excluded), are opened to participatory decision-making and integrate stakeholder knowledge^84,85^.

Synthetic biology can address global challenges, but the social consequences and benefits of the technology may be difficult to ascertain. For instance, the impetus for using synthetic biology to synthesise artemisinic acid, the precursor for the anti-malarial drug artemisinin, was due to increasing demand and widely varied annual production of *Artemisia annua*, the main source of artemisinin, which significantly affected the drug price and supply^86^. While synthetic production would have impacted the livelihood of the small-scale farmers in Asia and Africa who produced most of the *Artemisia annua* crop^87^, a lack of the drug would have resulted in increased fatalities from malaria, mostly in the developing world. After synthetic production was developed, agricultural production stabilised, the price dropped, and synthetic production was no longer commercially viable. Whether this was due to potential competition from the new source is unknown^88^. Regardless, the availability of a rapidly scalable process for synthesising artemisinin independent of agricultural issues, such as crop failure or disease, is beneficial.

## Governance challenges and changes

Innovations happen through convergence, which makes governance challenging and creates unanticipated regulatory gaps that need to be filled. Governance tools such as standards and regulatory sandboxes may address these gaps^89^. Additionally, the governance of GMOs, which forms a large part of the relevant regulatory framework around engineering biology and its products, varies widely around the world. These policy and regulatory frameworks have, in recent times, started to undergo significant shifts in locations such as the UK, EU and USA (Table 1).

**Table 1:** Comparison of some GMO regulatory steps

Regulatory steps	Explanation	UK	EU	USA
Pre-development risk assessment	Assessment of potential risks of R&D to human health and the environment	Conducted by developers, sometimes with assessment by SACGM, ACRE, HSE or other bodies; includes human health and environmental risks, containment measures etc.	Conducted by developers, member states and EFSA; includes human health and environmental risks, containment measures etc.	Conducted by developers, sometimes with assessment by USDA (e.g., APHIS), FDA, EPA; includes ecological and human health risks, containment measures etc.
R&D and/or trials	Research and development phase, including any necessary trials	Must comply with HSE regulations and legal conditions for trials	Must comply with local regulations and conditions, overseen by member states and EFSA	Must comply with federal regulations and may require Experimental Use Permits
Regulated product application	Application for product approval, assessing risks to human health and environment	Application includes detailed scientific data and risk assessments, reviewed by various committees depending on application	Application includes detailed scientific data and risk assessments, reviewed by member state competent authorities and EFSA	Application to USDA, FDA, or EPA, includes risk assessments and may involve GRAS determination
Scientific advisory committee review	Review by scientific advisory committees	May be reviewed by committees at DEFRA, FSA, HSE etc.; potentially assessed by reference laboratories; may request additional tests or information from developers	Reviewed by EFSA; potentially assessed by reference laboratories; may request additional tests or information from developers	Reviewed by relevant competent authority (USDA, FDA, EPA); may involve public consultation and scientific review
Public consultation and feedback	Period for public comments and feedback on the application	Consultation may take place during legislative process;	Public comments invited for 30–60 days; Member states develop emergency plans which can be consulted upon; compliance with transparency regulations	Public objections or complaints can be submitted within 30 days of an application’s publication
Decision to approve or request further info	Decision-making stage where approval is granted or further information requested	Decision to approve taken by ministers; may request further information before approval.	Vote by Standing Committee on Plants, Animals, Food and Feed (PAFF)	Decision by competent authority (USDA, FDA, EPA); may request further data or approve with conditions
Approval and conditions	Notification of approval and any associated legislative processes	Public notification of approval with or without conditions for distribution, sale, and use; compliance with inspections and other requirements	Public notification of approval with or without conditions for distribution, sale, and use; comply with inspections, reporting, and transparency requirements	Approval and registration with conditions for distribution, sale, and use; comply with inspections and reporting of adverse effects
Renewal of Authorisation	Process for renewing the authorisation after the initial approval period	Renew authorisation every 10 years	Renew GMO authorisation every 10 years	Renew permits and licences as required; comply with conditions and annual maintenance fees

Developers are challenging regulatory definitions for a range of reasons. Precise genetic engineering without introduction of foreign DNA, potentially limits risk. Prominent scientific and policy organisations have used these developments to argue for a differentiation between regulation designed to address Engineering Biology, and those introduced to control GMOs. Another key driver for this shift in regulation is that the GMO-relevant policy frameworks comprise several challenging features for developers. Both the UK and EU regulatory regimes contain both product- and process-focussed approaches, developed during the UK’s membership of the EU. Product-focused elements imply that GMOs are governed differently depending on their status as a novel food, animal feed, or whether they are plants, animals, or microorganisms. Process-focussed regulation assesses GMOs via separate regulatory pathways, which are distinct from pathways for other ‘risky’ organisms, by virtue of being produced through genetic modification^90^. There are also institutions and groups like advisory committees that only assess GM applications.

Further complicating matters, regulation in the EU is designed to apply across the bloc, but individual countries also have additional approaches to governing GMOs. For example, in Italy, there is what many perceive to be a de facto ban on cultivation of GMOs, and on the marketing of relevant ‘synthetic foods’. Each country individually evaluates applications to release or produce GMOs under the regulation and has their own approach to doing so. In the UK, Scotland, Northern Ireland, England and Wales have differing regulatory regimes in relation to GMOs in food and medicines. Northern Ireland’s regulatory regime is aligned to the EU approach. In Scotland, there are a range of specific regulations to govern GMOs. The GMOs (Deliberate Release) (Amendment) (England) Regulations 2022 only apply to England, but the Genetic Technology (Precision Breeding) Act 2023, which is broadly similar, applies to England, Scotland and Wales. This fragmented picture with overlapping rules introduces barriers to translating innovations into products and services. Regulatory diversity can also serve as an advantage, allowing different regions to experiment with various approaches. Successful policies or technologies implemented in one area can serve as models for others, promoting innovation and learning. This dynamic can lead to the adoption of best practices globally.

USA’s GMO governance is much more trait-focussed, or product-focussed, than the equivalent regimes in the UK and EU. However, there is fragmentation in how GMOs are regulated by different laws and institutions. For example, the FDA regulates foods and ingredients, agriculture, veterinary medicines, human drugs, biologics and medical devices. The EPA regulates pesticides and pollution in the environment and the USDA through APHIS focusses on protecting agriculture from pests and diseases. Through the Food Safety and Inspection Service (FSIS), the USDA oversees the safety and correct labelling of meat, poultry, and egg products, ensuring they meet federal standards for public health. This division of oversight means that different organisations issue permits and review risk assessments for different things, including whether a GMO is a ‘plant pest’ (APHIS), whether it is toxic to the environment (EPA), animals (APHA) or humans (FSIS and/or FDA).

Key changes in regulatory definitions are beginning to be developed around the terms ‘precision breeding’, ‘gene editing’, ‘GMO’ and ‘NGTs’. In the UK and USA, there are provisions for different regulatory treatment of some GMOs, which are known as ‘precision bred’ or ‘gene edited’, that do not involve the introduction of foreign DNA, or that could be deemed to have substantial equivalence to ‘natural’ products. In the EU, similar changes are in discussion to adjust the regulatory approach towards organisms produced through NGTs. Some argue these recent regulatory shifts simplify previous regimes and reflect a better understanding of the scientific landscape. Some consider such regulatory change to be based on fresh assessments of the risks of GMOs, including long-term risks that many expected when precautionary regulation was implemented, but are deemed not to have come to fruition in a global landscape of varying GM cultivation and use^91^. Others, however, challenge the jump from lab-based risk assessment methodologies to biological, ecological and toxicology claims, arguing that the push for revised regulatory frameworks is driven primarily by commercial interests^92^.

Overall, the field would benefit from shared terminologies and less disjointed regulatory process across geographies. This will facilitate the progression of discoveries and innovations at low technological readiness level toward deployable products and services, which today face lengthy GMO/engineering biology regulatory processes that need to be replicated across jurisdictions in the pursuit of wider markets.

## Future perspectives and challenges

Engineering biology is on the brink of transformative advances that promise to revolutionise environmental applications. Recent developments have been particularly promising in biosensing, bioremediation, bio-sequestration, pollution monitoring, and waste valorisation. Performance must meet real-world detection requirements, including high sensitivity and specificity, and high input/output dynamic ranges to cope with complex samples. We need field-deployable, biosafe, stable sensors with easy-to-read signals operable by non-specialists. As the field evolves, several challenges remain prominent. Scaling-up synthetic biological systems from lab to field poses significant hurdles, including maintaining the stability and efficacy of genetically engineered organisms in complex real-world environments. Additionally, while the cost of DNA synthesis has decreased, making high-throughput genetic engineering more accessible, ethical, regulatory, biosafety and biosecurity concerns continue to impose constraints on the broader application of these technologies. Regulatory frameworks should evolve to keep pace with technological advances to ensure safe and responsible application. The next steps for engineering biology for environmental applications will likely focus on refining the precision and efficiency of genetic tools. Continuing integration with computational modelling via, e.g., future multi-scale ecological scale digital-twins, AI and cyber-physical systems, referred to as bio-cyber-physical systems, will improve the design, optimisation, deployment and decommissioning of engineered organisms tailored for specific environmental tasks. Furthermore, as public awareness and engagement grow, there is potential for more inclusive and informed regulatory discussions, leading to more practical biosafety and ethical guidelines.

Looking forward, the integration of engineering biology with emerging technologies such as quantum computing and advanced materials science will open new pathways for innovation. For example, quantum computing could revolutionise the simulation of complex biological processes, leading to breakthroughs in enzyme design and metabolic pathway optimisation. The journey from laboratory research to real-world applications is fraught with both technical challenges and regulatory hurdles. Furthermore, scaling these innovations to a level where they can make a significant environmental impact requires technological readiness but also societal acceptance and robust regulatory frameworks. Therefore, while we can anticipate incremental advancements in the near term, the broader application of these revolutionary technologies could span several decades, depending on the pace of both scientific breakthroughs and corresponding policy developments.

## Supplementary information

Description of Additional Supplementary Information

Supplementary Data 1

## References

[1] Hassard, F. et al. Scaling-up engineering biology for enhanced environmental solutions. *ACS Synth. Biol.* **13**, 1586–1588 (2024). 38903005 10.1021/acssynbio.4c00292 PMC11197081

[2] National vision for engineering biology. *Department for Science, Innovation and Technology* (2023).

[3] Synthetic Biology Market. (Precedence Research, 2024).

[4] Chemla, Y., Sweeney, C. J., Wozniak, C. A. & Voigt, C. A. Design and regulation of engineered bacteria for environmental release. *Nat. Microbiol.* **10**, 281–300 (2025). 39905169 10.1038/s41564-024-01918-0

[5] Tellechea-Luzardo, J. et al. Linking engineered cells to their digital twins: a version control system for strain engineering. *ACS Synth. Biol.* **9**, 536–545 (2020). 32078768 10.1021/acssynbio.9b00400

[6] Brooks, S. M. & Alper, H. S. Applications, challenges, and needs for employing synthetic biology beyond the lab. *Nat. Commun.* **12**, 1390 (2021). 33654085 10.1038/s41467-021-21740-0 PMC7925609

[7] Freemont, P. S. et al. Engineering biology metrics and technical standards for the global bioeconomy. London, UK. (2024).

[8] El Karoui, M., Hoyos-Flight, M. & Fletcher, L. Future trends in synthetic biology - a report. *Front. Bioeng. Biotechol.* **7**, 175 (2019). 10.3389/fbioe.2019.00175 PMC6692427 31448268

[9] Wachter, G. K. A., Gallup, O., Bayne, J. & Horsfall, L. Synthetic biology landscape in the UK. *Biotechnol. Notes* **3**, 70–74 (2022). 39416441 10.1016/j.biotno.2022.07.002 PMC11446367

[10] Duncker, K. E., Holmes, Z. A. & You, L. C. Engineered microbial consortia: strategies and applications. *Microb. Cell Fact.* **20**, 211 (2021). 34784924 10.1186/s12934-021-01699-9 PMC8597270

[11] Fackler, N. et al. Stepping on the gas to a circular economy: accelerating development of carbon-negative chemical production from gas fermentation. *Annu. Rev. Chem. Biomol.* **12**, 439–470 (2021). 10.1146/annurev-chembioeng-120120-021122 33872517

[12] Agrawal, D. et al. Carbon emissions and decarbonisation: the role and relevance of fermentation industry in chemical sector. *Chem. Eng. J.* **475**, 146308 (2023).

[13] Sant’Anna, M. How green is sugarcane ethanol? *Rev. Econ. Stat.* **106**, 202–216 (2024).

[14] Bala, S. et al. Recent strategies for bioremediation of emerging pollutants: a review for a green and sustainable environment. *Toxics* **10**, 484 (2022). 36006163 10.3390/toxics10080484 PMC9413587

[15] de Lorenzo, V. Environmental galenics: large-scale fortification of extant microbiomes with engineered bioremediation agents. *Philos. Trans. R. Soc. B-Biol. Sci.* **377**, 20210395 (2022). 10.1098/rstb.2021.0395 PMC9234819 35757882

[16] French, K. E., Zhou, Z. R. & Terry, N. Horizontal ‘gene drives’ harness indigenous bacteria for bioremediation. *Sci. Rep.* **10**, 15091 (2020). 32934307 10.1038/s41598-020-72138-9 PMC7492276

[17] Janssen, D. B. & Stucki, G. Perspectives of genetically engineered microbes for groundwater bioremediation. *Environ. Sci. Proc. Imp.* **22**, 487–499 (2020). 10.1039/c9em00601j 32095798

[18] Ahuja, K. & Singh, S. Biosurfactants market size by product. *Glob. Market Insights* **564**, https://www.fortunebusinessinsights.com/biosurfactants-market-102761 (2020).

[19] Singh, P., Patil, Y. & Rale, V. Biosurfactant production: emerging trends and promising strategies. *J. Appl. Microbiol.* **126**, 2–13 (2019). 30066414 10.1111/jam.14057

[20] Desai, J. D. & Banat, I. M. Microbial production of surfactants and their commercial potential. *Microbiol. Mol. Biol. Rev.* **61**, 47 (1997). 9106364 10.1128/mmbr.61.1.47-64.1997 PMC232600

[21] Sundaram, T. et al. Advancements in biosurfactant production using agro-industrial waste for industrial and environmental applications. *Front. Microbiol.* **15**, 1357302 (2024). 10.3389/fmicb.2024.1357302 PMC10876000 38374917

[22] Chabhadiya, S., Acharya, D. K., Mangrola, A., Shah, R. & Pithawala, E. A. Unlocking the potential of biosurfactants: innovations in metabolic and genetic engineering for sustainable industrial and environmental solutions. *Biotechnol. Notes* **5**, 111–119 (2024). 39416688 10.1016/j.biotno.2024.07.001 PMC11446356

[23] Kong, W. T., Qian, Y. C., Stewart, P. S. & Lu, T. De novo engineering of a bacterial lifestyle program. *Nat. Chem. Biol.* **19**, 488 (2023). 36522463 10.1038/s41589-022-01194-1

[24] Ramos, J. L., de Lorenzo, V. & Lopez, P. Meeting report: microbes as safeguards of the environment. *Sustain. Microbiol.* **1**, qvae013 (2024). 10.1093/sumbio/qvae013 PMC13409297 42576915

[25] Zhu, N. L., Zhang, B. & Yu, Q. L. Genetic engineering-facilitated coassembly of synthetic bacterial cells and magnetic nanoparticles for efficient heavy metal removal. *ACS Appl. Mater. Interfaces* **12**, 22948–22957 (2020). 32338492 10.1021/acsami.0c04512

[26] Nguyen, P. Q. et al. Wearable materials with embedded synthetic biology sensors for biomolecule detection. *Nat. Biotechnol.* **39**, 1366 (2021). 34183860 10.1038/s41587-021-00950-3

[27] Akyildiz, I. F., Pierobon, M., Balasubramaniam, S. & Koucheryavy, Y. The internet of bio-nano things. *IEEE Commun. Mag.* **53**, 32–40 (2015).

[28] Helmy, M., Smith, D. & Selvarajoo, K. Systems biology approaches integrated with artificial intelligence for optimized metabolic engineering. *Metab. Eng. Commun.* **11**, e00149 (2020). 33072513 10.1016/j.mec.2020.e00149 PMC7546651

[29] Kim, D. S., Moreno-Cabezuelo, J. A., Schulz, E. N., Lea-Smith, D. J. & Sagaram, U. S. Recent advances in engineering fast-growing cyanobacterial species for enhanced CO2 fixation. *Front. Clim.* **6**, 1412232 (2024).

[30] Teworte, S., Malci, K., Walls, L. E., Halim, M. & Rios-Solis, L. Recent advances in fed-batch microscale bioreactor design. *Biotechnol. Adv.* **55**, 107888 (2022). 10.1016/j.biotechadv.2021.107888 34923075

[31] Oks, S. J. et al. Cyber-physical systems in the context of industry 4.0: a review, categorization and outlook. *Inform. Syst. Front.* **26**, 1731–1772 (2022).

[32] Voigt, C. A. Synthetic biology 2020-2030: six commercially-available products that are changing our world. *Nat. Commun.* **11**, 6379 (2020). 33311504 10.1038/s41467-020-20122-2 PMC7733420

[33] Jessop-Fabre, M. M. & Sonnenschein, N. Improving reproducibility in synthetic biology. *Front. Bioeng. Biotechnol.* **7** (2019). 10.3389/fbioe.2019.00018 PMC6378554 30805337

[34] Zhu, X. J. et al. Engineered Bacillus subtilis Biofilm@Biochar living materials for in-situ sensing and bioremediation of heavy metal ions pollution. *J. Hazard. Mater.* **465**, 133119 (2024). 10.1016/j.jhazmat.2023.133119 38134689

[35] Kim, H. S. et al. Biocomposite thermoplastic polyurethanes containing evolved bacterial spores as living fillers to facilitate polymer disintegration. *Nat. Commun.* **15**, 3338 (2024). 38688899 10.1038/s41467-024-47132-8 PMC11061138

[36] Tang, T. C. et al. Materials design by synthetic biology. *Nat. Rev. Mater.* **6**, 332–350 (2021).

[37] George, D. R. et al. A bumpy road ahead for genetic biocontainment. *Nat. Commun.* **15**, 650 (2024). 38245521 10.1038/s41467-023-44531-1 PMC10799865

[38] Venkateswaran, N., Senthil Kumar, S., Diwakar, G., Gnanasangeetha, D. & Boopathi, S. In: *Applications of Synthetic Biology in Health, Energy, and Environment* (ed. M. Arshad) 360–384 (IGI Global, 2023).

[39] Clarke, L. & Kitney, R. Developing synthetic biology for industrial biotechnology applications. *Biochem. Soc. T* **48**, 113–122 (2020). 10.1042/BST20190349 PMC7054743 32077472

[40] Chubukov, V., Mukhopadhyay, A., Petzold, C. J., Keasling, J. D. & Martín, H. G. Synthetic and systems biology for microbial production of commodity chemicals. *Npj Syst. Biol. Appl.* **2**, 16009 (2016). 10.1038/npjsba.2016.9 PMC5516863 28725470

[41] Chesbrough, H. Open innovation: where we’ve been and where we’re going. *Res. Technol. Manag.* **55**, 2027 (2012).

[42] Trump, B. D. Synthetic biology regulation and governance: lessons from TAPIC for the United States, European Union, and Singapore. *Health Policy* **121**, 1139–1146 (2017). 28807332 10.1016/j.healthpol.2017.07.010

[43] Escalante, A. E., Rebolleda-Gómez, M., Benítez, M. & Travisano, M. Ecological perspectives on synthetic biology: insights from microbial population biology. *Front. Microbiol.* **6**, 143 (2015). 10.3389/fmicb.2015.00143 PMC4341553 25767468

[44] Mekonnen, B. A., Aragaw, T. A. & Genet, M. B. Bioremediation of petroleum hydrocarbon contaminated soil: a review on principles, degradation mechanisms, and advancements. *Front. Env. Sci. Switz.* **12** (2024).

[45] Thompson, I. P., van der Gast, C. J., Ciric, L. & Singer, A. C. Bioaugmentation for bioremediation: the challenge of strain selection. *Environ. Microbiol.* **7**, 909–915 (2005). 15946288 10.1111/j.1462-2920.2005.00804.x

[46] Hanson, A. D. & de Lorenzo, V. Synthetic biology-high time to deliver? *ACS Synth. Biol.* **12**, 1579–1582 (2023). 37322887 10.1021/acssynbio.3c00238 PMC10278163

[47] Yang, J., Kim, B., Kim, G. Y., Jung, G. Y. & Seo, S. W. Synthetic biology for evolutionary engineering: from perturbation of genotype to acquisition of desired phenotype. *Biotechnol. Biofuels* **12**, 113 (2019). 10.1186/s13068-019-1460-5 PMC6506968 31086565

[48] Rao, G. S., Jiang, W. J. & Mahfouz, M. Synthetic directed evolution in plants: unlocking trait engineering and improvement. *Synth. Biol.* **6**, 1–6 (2021). 10.1093/synbio/ysab025 PMC8434914 34522785

[49] Jeong, S. H., Lee, H. J. & Lee, S. J. Recent advances in CRISPR-Cas technologies for synthetic biology. *J. Microbiol.* **61**, 13–36 (2023). 36723794 10.1007/s12275-022-00005-5 PMC9890466

[50] Beeckman, D. S. A. & Rüdelsheim, P. Biosafety and biosecurity in containment: a regulatory overview. *Front. Bioeng. Biotechnol.* **8**, 650 (2020). 10.3389/fbioe.2020.00650 PMC7348994 32719780

[51] Lensch, A. et al. Safety aspects of microorganisms deliberately released into the environment. *EFB Bioecon. J.* **4**, 100061 (2024).

[52] Mandell, D. J. et al. Biocontainment of genetically modified organisms by synthetic protein design (vol 518, pg 55, 2015). *Nature* **527**, 264–264 (2015). 25607366 10.1038/nature14121 PMC4422498

[53] Schmidt, M. & Kubyshkin, V. How to quantify a genetic firewall? A polarity-based metric for genetic code engineering. *Chembiochem* **22**, 1268–1284 (2021). 33231343 10.1002/cbic.202000758 PMC8049029

[54] Wilson, D. J. NIH guidelines for research involving recombinant DNA molecules. *Acc. Res.* **3**, 177–185 (1993). 10.1080/08989629308573848 11652293

[55] Gallagher, R. R., Patel, J. R., Interiano, A. L., Rovner, A. J. & Isaacs, F. J. Multilayered genetic safeguards limit growth of microorganisms to defined environments. *Nucleic Acids Res.* **43**, 1945–1954 (2015). 25567985 10.1093/nar/gku1378 PMC4330353

[56] Hirota, R. et al. A novel biocontainment strategy makes bacterial growth and survival dependent on phosphite. *Scientific Reports* **7**, 44748 (2017). 10.1038/srep44748 PMC5357788 28317852

[57] Cai, Y. Z. et al. Intrinsic biocontainment: Multiplex genome safeguards combine transcriptional and recombinational control of essential yeast genes. *Proc. Natl Acad. Sci. USA* **112**, 1803–1808 (2015). 25624482 10.1073/pnas.1424704112 PMC4330768

[58] Arnolds, K. L. et al. Biotechnology for secure biocontainment designs in an emerging bioeconomy. *Curr. Opin. Biotechnol.* **71**, 25–31 (2021). 34091124 10.1016/j.copbio.2021.05.004

[59] Maull, V. & Sole, R. Network-level containment of single-species bioengineering. *Philos. Trans. R. Soc. B-Biol. Sci.* **377**, 20210396 (2022). 10.1098/rstb.2021.0396 PMC9234816 35757875

[60] Maull, V. & Solé, R. Biodiversity as a firewall to engineered microbiomes for restoration and conservation. *Roy. Soc. Open Sci.* **11** (2024). 10.1098/rsos.231526 PMC11296081 39100153

[61] Wang, F. & Zhang, W. Synthetic biology: recent progress, biosafety and biosecurity concerns, and possible solutions. *J. Biosaf. Biosecur.* **1**, 22–30 (2019).

[62] Heider, D., Pyka, M. & Barnekow, A. DNA watermarks in non-coding regulatory sequences. *BMC Res. Notes* **2**, 125 (2009). 19583865 10.1186/1756-0500-2-125 PMC2713970

[63] More, S. et al. Evaluation of existing guidelines for their adequacy for the microbial characterisation and environmental risk assessment of microorganisms obtained through synthetic biology. *EFSA J* **18**, e06301 (2020). 10.2903/j.efsa.2020.6263 PMC7592124 33144886

[64] Nielsen, A. A. K. & Voigt, C. A. Deep learning to predict the lab-of-origin of engineered DNA. *Nat. Commun.* **9**, 3135 (2018). 10.1038/s41467-018-05378-z PMC6081423 30087331

[65] Kalvapalle, P. B. et al. Information storage across a microbial community using universal RNA barcoding. *Nat. Biotechnol.* https://www.biorxiv.org/content/10.1101/2023.04.16.536800v1 (2025). 10.1038/s41587-025-02593-0 40102641

[66] de Lorenzo, V., Krasnogor, N. & Schmidt, M. For the sake of the bioeconomy: define what a synthetic biology chassis is! *N. Biotechnol.* **60**, 44–51 (2021). 32889152 10.1016/j.nbt.2020.08.004

[67] Tellechea-Luzardo, J. et al. Versioning biological cells for trustworthy cell engineering. *Nat. Commun.* **13**, 765 (2022). 10.1038/s41467-022-28350-4 PMC8828774 35140226

[68] Sabary, O., Kiah, H. M., Siegel, P. H. & Yaakobi, E. Survey for a decade of coding for DNA storage. *IEEE T Mol. Biol. Mult.* **10**, 253–271 (2024).

[69] Berezin, C. T., Peccoud, S., Kar, D. M. & Peccoud, J. Cryptographic approaches to authenticating synthetic DNA sequences. *Trends Biotechnol.* **42**, 1002–1016 (2024). 38418329 10.1016/j.tibtech.2024.02.002 PMC11309913

[70] Wittmann, B. J. et al. Toward AI-resilient screening of nucleic acid synthesis orders: process, results, and recommendations. *bioRxiv*, https://www.biorxiv.org/content/10.1101/2024.12.02.626439v1 (2024).

[71] GOV.UK. Joint Statement: US-UK Strategic Dialogue on biological security. (2024).

[72] GOV.UK. UK screening guidance on synthetic nucleic acids for users and providers. Department for science, innovation & technology (2024).

[73] Services, U. S. D. o. H. H. Screening framework guidance for providers and users of synthetic nucleic acids. (2023).

[74] IBBIS. A free, open-source, globally-available tool for synthesis screening, https://ibbis.bio/our-work/common-mechanism/ (2024).

[75] SecureDNA. Safeguarding DNA synthesis, https://securedna.org/ (2024).

[76] Macnaghten, P., Owen, R. & Jackson, R. Synthetic biology and the prospects for responsible innovation. *Essays Biochem.* **60**, 347–355 (2016). 27903822 10.1042/EBC20160048

[77] Pauwels, E. Public understanding of synthetic biology. *BioScience* **63**, 79–89 (2013).

[78] Stirling, A., Hayes, K. R. & Delborne, J. Towards inclusive social appraisal: risk, participation and democracy in governance of synthetic biology. *BMC Proc.* **12**, 15 (2018). 30079106 10.1186/s12919-018-0111-3 PMC6069769

[79] Sharp, L. Reconnecting people and water: public engagement and sustainable urban water management. *Landsc. Arch. Mag.* **107**, 138–138 (2017).

[80] van der Horst, D. & Vermeylen, S. Spatial scale and social impacts of biofuel production. *Biomass Bioenerg.* **35**, 2435–2443 (2011).

[81] Ribeiro, B. E. Beyond commonplace biofuels: social aspects of ethanol. *Energy Policy* **57**, 355–362 (2013).

[82] Bernstein, M. J., Franssen, T., Smith, R. D. J. & de Wilde, M. The European Commission’s Green Deal is an opportunity to rethink harmful practices of research and innovation policy. *Ambio* **52**, 508–517 (2023). 36324020 10.1007/s13280-022-01802-3 PMC9849660

[83] Wickson, F. et al. Addressing socio-economic and ethical considerations in biotechnology governance: the potential of a new politics of care. *Food Ethics* **1**, 193–199 (2017).

[84] Raman, S. et al. Integrating social and value dimensions into sustainability assessment of lignocellulosic biofuels. *Biomass Bioenerg.* **82**, 49–62 (2015). 10.1016/j.biombioe.2015.04.022 PMC4643753 26664147

[85] Sevigné-Itoiz, E., Mwabonje, O., Panoutsou, C. & Woods, J. Life cycle assessment (LCA): informing the development of a sustainable circular bioeconomy? *Philos. T. R. Soc. A* **379**, 20200352 (2021). 10.1098/rsta.2020.0352 PMC8326828 34334023

[86] Van Noorden, R. Demand for malaria drug soars. *Nature* **466**, 672–673 (2010). 20686539 10.1038/466672a

[87] Dalziell, J. & Rogers, W. Are the ethics of synthetic biology fit for purpose? A case study of artemisinin. *Proc. IEEE* **110**, 511–517 (2022).

[88] Peplow, M. Synthetic biology’s first malaria drug meets market resistance. *Nature* **530**, 389–390 (2016). 26911755 10.1038/530390a

[89] Pei, L., Garfinkel, M. & Schmidt, M. Bottlenecks and opportunities for synthetic biology biosafety standards. *Nat. Commun.* **13**, 2175 (2022). 10.1038/s41467-022-29889-y PMC9023567 35449163

[90] Lezaun, J. Creating a new object of government: making genetically modified organisms traceable. *Soc. Stud. Sci.* **36**, 499–531 (2006).

[91] Reforming the Governance of Genetic Technologies. *Policy Brief by the Regulatory Horizons Council*, https://assets.publishing.service.gov.uk/media/62bef4068fa8f578be2f7b71/regulatory_horizons_council_policy_brief_on_genetic_technologies.pdf (2022).

[92] Hilbeck, A., Meyer, H., Wynne, B. & Millstone, E. GMO regulations and their interpretation: how EFSA’s guidance on risk assessments of GMOs is bound to fail. *Environ. Sci. Europe* **32**, 54 (2020).

