# Supplementary material for: Engineering biology applications for environmental solutions: potential and challenges
Source: Nat Commun. 2025 Apr 14;16:3538. doi: 10.1038/s41467-025-58492-0 (PMC11997111; doi:10.1038/s41467-025-58492-0)
Supplement: Supplementary file 1 — Description of Additional Supplementary Information [file 41467_2025_58492_MOESM1_ESM.docx]

**Supplementary Data Legend**

**Supplementary Data 1: Companies commercialising engineering biology and bioremediation.**
